# Increased contractility affects left ventricular kinetic energy in pulmonary hypertension

**DOI:** 10.14814/phy2.70563

**Published:** 2025-09-12

**Authors:** E. Bergström, K. Pola, B. Kjellström, J. Töger, P. M. Arvidsson, M. Carlsson, G. Rådegran, H. Arheden, E. Ostenfeld

**Affiliations:** ^1^ Department of Clinical Sciences Lund, Clinical Physiology Lund University, Skåne University Hospital Lund Sweden; ^2^ Department of Clinical Sciences Lund, Cardiology, Section of Heart Failure and Valvular Disease Lund University, Skåne University Hospital Lund Sweden

**Keywords:** 4D flow, cardiac hemodynamics, contractility, kinetic energy, pulmonary hypertension

## Abstract

Precapillary pulmonary hypertension (PH) is characterized by increased pulmonary vascular resistance (PVR), with progressively altered right (RV) and left ventricular (LV) hemodynamics and function. Kinetic energy (KE) from 4D flow cardiovascular magnetic resonance (CMR) is a measure of intracardiac hemodynamics. In this observational case–control study, we investigate physiological mechanisms influencing RV‐KE and LV‐KE in PH. Twenty PH patients and 12 healthy controls underwent CMR including cine images and 4D flow. LV contractility was derived from noninvasive pressure‐volume loops, and PVR from right heart catheterization. RV‐KE and LV‐KE were computed for systole, early and late diastolic filling, and indexed to stroke volume (SV). Systolic RV‐KE did not differ between patients and controls. In patients, systolic RV‐KE was associated with RV‐SV but not with PVR, suggesting that the RV may still be able to compensate for the increased afterload. Systolic LV‐KE indexed to LV‐SV, LV contractility, and heart rate were all higher in patients than controls, suggesting sympathetic upregulation as a possible driving mechanism behind increased systolic LV‐KE. LV contractility was negatively associated with systolic LV‐KE and LV‐SV. Late filling KE was increased in both ventricles in patients, suggesting an enhanced importance of the atrial kick to the filling of both ventricles.

## Graphical abstract

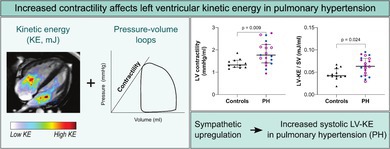

## 1 INTRODUCTION

Precapillary pulmonary hypertension (PH) is characterized by remodeling of the pulmonary arteries, entailing flow obstruction in the pulmonary circulation, and is associated with a poor prognosis if right heart failure occurs (Galiè et al., 2016). Increased right ventricular (RV) volumes and decreased RV ejection fraction (EF) as well as decreased left ventricular (LV) volumes are prognostic markers of outcome in PH (Dong et al., 2020; Henein et al., 2017; Humbert et al., 2022; van Wolferen et al., 2007). However, the prognostic ability of RVEF has been debated (Ostenfeld & Kjellström, 2020), and LV longitudinal function can be reduced despite preserved LVEF (Bredfelt et al., 2018; Dong et al., 2020; Lindholm et al., 2022; Ostenfeld & Kjellström, 2020). This suggests a presence of biventricular dysfunction, beyond what is indicated by reduced EF. Improved and refined methods for detection of biventricular dysfunction are warranted.

Kinetic energy (KE) is the energy contained in the blood due to motion and may be an indicator of biventricular dysfunction (Bolger et al., 2007; Han et al., 2015). KE of intraventricular blood flow can be computed using four‐dimensional (4D) flow by cardiovascular magnetic resonance imaging (CMR) (Arvidsson et al., 2013; Bissell et al., 2023; Carlsson et al., 2012). Using CMR, it has been shown that intraventricular KE is affected by age and sex (Wong et al., 2016; Zhao et al., 2023), and is altered in several conditions such as left ventricular heart failure (Arvidsson et al., 2022; Kanski, Arvidsson, et al., 2015), dilated cardiomyopathy (Eriksson et al., 2013), repaired tetralogy of Fallot (Sjoberg et al., 2018), and after myocardial infarction (Ben‐Arzi et al., 2022; Garg et al., 2018). While ventricular KE has been previously quantified in PH (Han et al., 2015; Zhao et al., 2022), the physiological determinants of variations in RV‐KE and LV‐KE in PH are not well understood.

Therefore, the purpose of this study was to investigate the association between hemodynamic state and RV‐KE and LV‐KE in patients with PH compared to healthy controls.

## 2 METHODS

This prospective, observational, case–control study is reported according to STROBE guidelines (von Elm et al., 2007).

### 2.1 Study population

We prospectively included consecutive patients with suspected or confirmed PH referred for clinical CMR investigation at our tertiary PH center (Skåne University Hospital in Lund, Sweden) between 2016 and 2021. Inclusion criteria were as follows: Patients aged ≥16 years with a confirmed diagnosis of PH owing to idiopathic or hereditary pulmonary arterial hypertension (PAH), pulmonary hypertension associated with connective tissue disease, or chronic thromboembolic pulmonary hypertension (CTEPH), as all three patient groups may be expected to have similar intracardiac pressure conditions. Patients with atrial fibrillation, cardiac shunts, and/or inadequate CMR image quality were excluded.

The control group consisted of healthy volunteers (*n* = 12) matched for age and sex on the group level. Controls were never‐smokers, had no known cardiovascular or systemic disease including systemic hypertension, had no cardiovascular treatment, and had no pathological findings on ECG or CMR.

Noninvasive systemic blood pressure was measured with a brachial cuff and sphygmomanometer at the time of the CMR examination.

All participants gave written informed consent to participate. All patients and controls have previously been included in a study from our research group (Pola et al., 2022). The Regional Ethical Committee in Lund, Sweden has approved the project. The study complies with the Declaration of Helsinki.

### 2.2 Right heart catheterization

PH diagnosis was confirmed by right heart catheterization (Galiè et al., 2004, 2016). A triple‐lumen Swan‐Ganz catheter was inserted in a jugular vein under local anesthesia. Pressure measurements were obtained in a supine position and during free breathing. Measurements of right atrial pressure, pulmonary arterial pressure, and pulmonary artery wedge pressure were acquired as the mean over several heartbeats. Pulmonary vascular resistance (PVR) was computed as (mean pulmonary arterial pressure−pulmonary artery wedge pressure)/cardiac output. Precapillary PH was defined as a mean pulmonary arterial pressure ≥25 mmHg, pulmonary artery wedge pressure ≤15 mmHg, and PVR >3 Wood Units, according to ESC/ERS guidelines current at the time of diagnosis (Galiè et al., 2004, 2016).

### 2.3 Image acquisition and processing

Cardiovascular magnetic resonance imaging was acquired in a supine position using a 1.5T MAGNETOM Aera (Siemens Healthineers, Forchheim, Germany) magnetic resonance imaging scanner. Balanced steady‐state free precession cine images were acquired during end‐expiratory breath‐hold in three standard long‐axis views and in a short‐axis stack covering the ventricles. Typical parameters for the short‐axis stack were as follows: Repetition time 41 ms, echo time 1.1 ms, flip angle 62 degrees, spatial resolution (acquired) 1.8 × 1.9 mm^2^, spatial resolution (reconstructed) 1.1 × 1.1 mm^2^, slice thickness 8 mm, temporal resolution (reconstructed) 34 ms.

ECG‐triggered, three‐dimensional, time‐resolved 4D flow images were acquired covering the entire heart and proximal great vessels, using a research sequence (Carlsson et al., 2011; Dyverfeldt et al., 2015; Kanski, Toger, et al., 2015; Töger et al., 2016). Typical parameters of the 4D flow sequence were: temporal resolution (acquired) 46 ms, spatial resolution (acquired) 3 mm isotropic, acceleration methods GRAPPA factor 3–4 (3 × 1 or 2 × 2, phase×slice encoding direction) and partial Fourier 6/8 in both phase encoding and slice encoding directions (Pola et al., 2022). Detailed sequence parameters are shown in Table S1.

Two‐dimensional flow (2D flow) phase contrast images were acquired perpendicular to the ascending aorta and pulmonary artery. Typical parameters were as follows: Repetition time 9.8 ms, echo time 2.67 ms, flip angle 20 degrees, spatial resolution (reconstructed) 1.5 mm, slice thickness 5 mm, temporal resolution (reconstructed) 24 ms, and VENC 200 cm/s.

### 2.4 Image analysis

All CMR images were analyzed using the freely available software Segment v2.2 R7052 for KE analysis and planimetry, and v3.3 R10057 (Medviso AB, Sweden) (Heiberg et al., 2010) for noninvasive pressure‐volume (PV) loops. Delineations of all images were performed by one observer (EB or KP) with a second observer (EO, EACVI CMR level 3 with >15 years of experience) as adjudicator.

**FIGURE 1:**
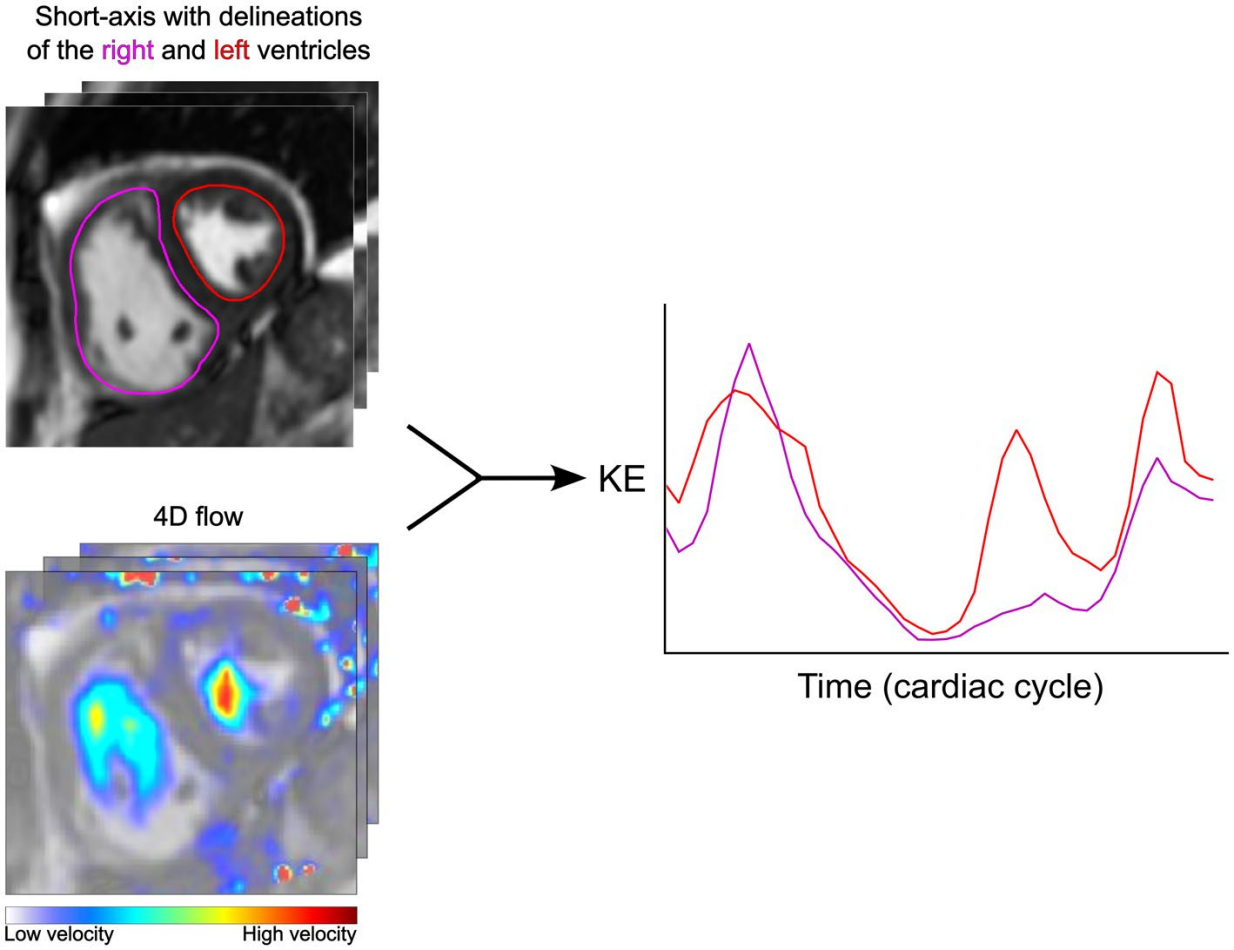
Computation of kinetic energy (KE). Left top: Delineations of the right (magenta) and left (red) ventricles in a patient with precapillary pulmonary hypertension, and left bottom: Velocity data from 4D flow. Right: Kinetic energy data from both ventricles and for the entire cardiac cycle.

Endocardial borders of both ventricles were manually delineated in cine short‐axis images in all slices and time frames (Figure 1). Trabeculations and papillary muscles were included in the intraventricular blood pool (Schulz‐Menger et al., 2020). Epicardial borders of both ventricles were manually delineated in all slices at end diastole and end systole. End‐diastolic and end‐systolic volumes, as well as stroke volume (SV) and EF, were computed for both ventricles. Myocardial mass was computed as the difference between epicardial and endocardial volumes, multiplied by an assumed myocardial density of 1.05 g/mL. Myocardial mass, end‐diastolic and end‐systolic volumes, as well as SV, were reported in absolute values and indexed to body surface area.

Velocity aliasing correction was semiautomatically performed on the 4D flow using phase‐unwrapping (Yang et al., 1996). Background phase errors were corrected with fitting to stationary tissue, using either first or second order correction based on visual assessment (Busch et al., 2017; Gatehouse et al., 2010). The spatial orientation of the 4D flow data was manually aligned to the short‐axis cine images when needed. The time‐resolved delineations from cine short‐axis images were interpolated and superimposed onto the 4D flow image stacks.

**FIGURE 2:**
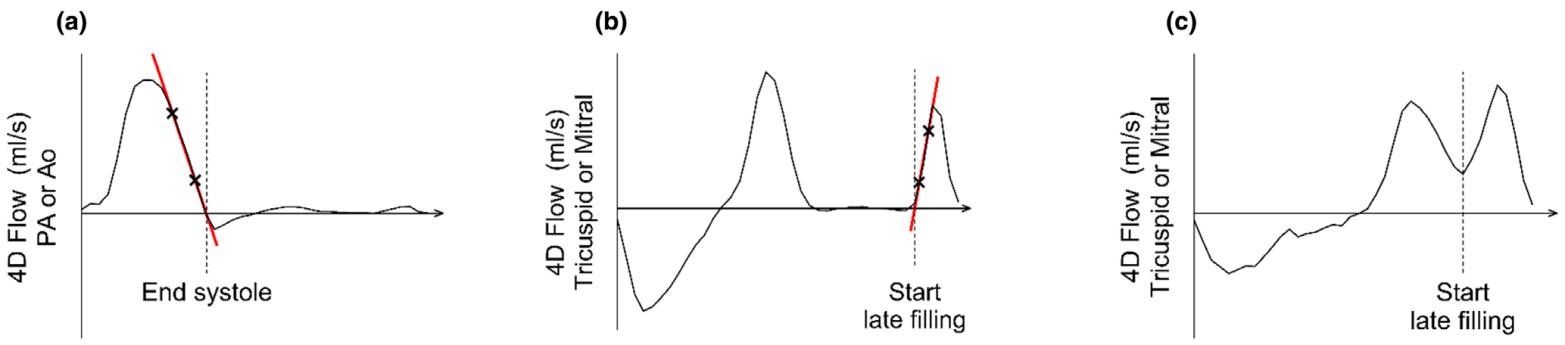
Definition of cardiac phases based on 4D flow. (a) 4D flow curve from the pulmonary artery (PA) or aorta (Ao) was used to define end systole in both ventricles. The red line passes through 75% and 25% of the maximum amplitude of the systolic downslope and crosses the x‐axis at the time of end systole. (b) 4D flow curve from transtricuspid or transmitral flow was used to define the start of late filling of both ventricles. (c) Transtricuspid or transmitral flow curve when the flow did not reach zero during diastasis. The start of late filling was defined from the lowest point of the flow between early and late filling.

The timing of each cardiac phase was defined using 4D flow as previously described (Toger et al., 2012), and a detailed description of the method is provided in Appendix S1. In short, end diastole for KE analysis was defined as the first time frame in the 4D flow data. For end systole, a line was extrapolated from the systolic downslope, going through two points at 75% and 25% of maximum amplitude. End systole was defined where the extrapolated line reached zero (Figure 2a). Similarly, the transition from early to late filling during diastole was defined using transtricuspid and transmitral flow curves at the approximate level of the valve leaflets (Figure 2b). In the cases where the transtricuspid or transmitral curves did not reach zero, the lowest point of the flow curve between early and late filling defined the start of late filling diastole (Figure 2c). In cases of fusion with no nadir between early and late filling, only total diastolic KE was reported, where early and late filling are treated as one continuous phase. Total diastolic KE (early and late filling treated as one continuous phase) is reported from all participants, while early and late filling values are only reported where the two phases could be separated.

KE of both ventricular blood pools was computed over the entire cardiac cycle using the 4D flow velocities. KE was computed for each voxel separately as KE = ½ *mv* ^2^, where *m* is the mass of the blood assuming a density of 1.05 g/mL (Trudnowski & Rico, 1974), and *v* is velocity. Ventricular KE was then computed for each point in time as the KE sum of all voxels within the ventricular delineations.

KE was computed for each cardiac phase as peak and mean values and was indexed to SV from planimetry to account for the blood volume leaving the ventricle. All reported KE values in the main results are peak KE unless otherwise stated. Additionally, KE indexed to end‐diastolic volume was calculated and reported in Appendix S1. Tricuspid regurgitation fraction was calculated from the difference in planimetric and 2D flow RV‐SV, divided by the planimetric RV‐SV. LV contractility was computed from noninvasive pressure‐volume loops using a previously described and validated method (Arvidsson et al., 2023; Seemann et al., 2019). In short, heart rate and LV volumes from cine short‐axis images and noninvasive brachial blood pressure were used as input data into a model based on the elastance of the LV, which generated noninvasive pressure‐volume loops (Seemann et al., 2019). Contractility was computed as the slope of the end‐systolic pressure‐volume relationship (Figure S1).

### 2.5 Statistical analysis

Statistical analysis was performed using Prism v9.4.0 (GraphPad Software, La Jolla, California, USA). Normal distribution was visually assessed using histograms. Continuous data is expressed as median and interquartile range [IQR] owing to non‐Gaussian distribution. Categorical data is expressed as absolute numbers and proportion in percentage. The Mann–Whitney *U* test and Fisher's exact test were used for comparisons between groups. Linear regressions were performed for association analysis of KE versus PVR and contractility, and the normality of residuals was assessed using the D'Agostino–Pearson omnibus K2 test. PVR was included in the regression analyses when right heart catheterization and CMR were both performed within a 4‐week time period, and no changes in cardiovascular medications or clinical status had occurred during that time. A two‐tailed *p* value <0.05 was considered statistically significant.

## 3 RESULTS

In total, 44 patients were investigated for PH with CMR and 4D flow during the inclusion period. Of these, 24 patients were excluded due to: Inadequate 4D flow quality (*n* = 7), missing slices in cine short axis images (*n* = 1), or not meeting the diagnostic inclusion criteria (*n* = 16). Thus, 20 patients with PH (median and [IQR], age 69 [18] years, 16 women) and 12 age‐matched controls (62 [6] years, nine women) were included in the final analysis (Table 1). Catheterization measurements from all patients are reported in Table 2. A majority of the patients (*n* = 13/20, 65%) had PAH and the remaining had CTEPH (7/20, 35%). Most patients were newly diagnosed and treatment naive, while 30% were treated with PH‐specific medication at the time of CMR. Patients had cardiovascular comorbidities typical for a PH cohort, and hence received other cardiovascular treatments (Table 2). Six (30%) patients had chronic obstructive pulmonary disease (COPD) or emphysema. COPD and emphysema were clinically assessed as not being the predominant cause of PH in concordance with ESC/ERS guidelines (Galiè et al., 2016; Humbert et al., 2022).

**TABLE 1:** Participant characteristics.

	Controls (*n* = 12)	PH (*n* = 20)	*p* Value
Age (years)	62 [6]	69 [18]	0.092
Women, *n* (%)	9 (75)	16 (80)	>0.999
Body mass index (kg/m^2^)	24.8 [3.1]	26.1 [6.7]	0.096
Body surface area (m^2^)	1.8 [0.2]	1.8 [0.3]	0.962
Noninvasive systemic blood pressure (mmHg)			
Systolic	122 [17]^a^	138 [28]^b^	0.050
Diastolic	77 [14]^a^	87 [18]^b^	0.083

*Note*: Data is expressed as median and interquartile range [IQR] or absolute numbers and proportion (%). Two‐tailed *p* values <0.05 were considered statistically significant.

Abbreviation: PH, precapillary pulmonary hypertension.

^a^ *n* = 11.

^b^ *n* = 19.

**TABLE 2:** Clinical variables.

	PH (*n* = 20)
PH subgroups	
PAH etiology, *n* (%)	13 (65%)
Idiopathic/hereditable PAH (*n*)	8
Associated PAH (connective tissue disease) (*n*)	5
Chronic thromboembolic pulmonary hypertension, *n* (%)	7 (35%)
Incidental cases at CMR^a^, *n* (%)	14 (70%)
NT‐proBNP (ng/L)^b^	475 [1332]
Right heart catheterization	
Time between CMR and right heart catheterization (days)	9 [63]
Systolic pulmonary arterial pressure (mmHg)	74 [31]
Mean pulmonary arterial pressure (mmHg)	46 [19]
Right ventricular pressure (mmHg)	
Systolic	73 [33]
Diastolic	0 [5]
End‐diastolic	9 [5]
Mean right atrial pressure (mmHg)	6 [5]
Pulmonary artery wedge pressure (mmHg)	7 [3]
Pulmonary vascular resistance (Wood units)	8 [6]
Systemic vascular resistance (Wood units)	19 [13]
Comorbidities	
Chronic obstructive pulmonary disease/emphysema, *n* (%)	6 (30%)
Diabetes, *n* (%)	5 (25%)
Ischemic heart disease, *n* (%)	2 (10%)
Systemic hypertension, *n* (%)	9 (45%)
Right bundle branch block, complete or incomplete, *n* (%)	2 (10%)
Left bundle branch block, *n* (%)	1 (5%)
Medication at CMR	
PH‐specific medication	
Single therapy, *n* (%)	2 (10%)
Dual therapy, *n* (%)	2 (10%)
Triple therapy, *n* (%)	2 (10%)
Other cardiovascular medications	
Calcium channel blockers (due to reasons other than PH), *n* (%)	6 (30%)
ACEi/ARB/ARNI, *n* (%)	8 (40%)
Betablockers, *n* (%)	5 (25%)
Diuretics, *n* (%)	8 (40%)

*Note*: Data is expressed as median and interquartile range [IQR] or absolute numbers and proportion (%). PH‐specific medication includes endothelin receptor antagonist, phosphodiesterase 5 inhibitor, prostaglandin analogue, selective prostacyclin receptor agonist, and soluble guanylate cyclase stimulator.

Abbreviations: ACEi, angiotensin‐converting enzyme inhibitor; ARB, angiotensin receptor blocker; ARNI, angiotensin receptor‐neprilysin inhibitor; CMR, cardiovascular magnetic resonance imaging; NT‐proBNP, N‐terminal pro b‐type natriuretic peptide; PAH, pulmonary arterial hypertension; PH, precapillary pulmonary hypertension.

^a^ Cases where CMR was performed in conjunction with diagnosing the patient with PH.

^b^ *n* = 19.

Cardiac volumes and functional measures from CMR are presented in Table 3. RV volumes and mass were larger, tricuspid regurgitation fraction higher, and RVEF lower in patients than controls. LV volumes indexed for body surface area were lower in patients compared to controls, while there was no difference in LVEF or cardiac index between groups. Patients had higher heart rates than controls.

**TABLE 3:** CMR variables.

		Controls (*n* = 12)	PH (*n* = 20)	*p* Value
	Heart rate (bpm)	69 [10]	76 [15]	**0.032**
RV	EDV (mL)	151 [41]	194 [84]	**0.036**
	EDVi (mL/m^2^)	84 [15]	110 [44]	**0.013**
	ESV (mL)	73 [21]	121 [78]	**0.007**
	ESVi (mL/m^2^)	37 [7]	67 [45]	**0.002**
	SV (mL)	82 [18]	76 [42]	0.597
	SVi (mL/m^2^)	45 [12]	42 [17]	0.604
	EF (%)	54 [6]	43 [18]	**0.0008**
	Tricuspid regurgitation fraction (%)	5 [9]	15 [11]	**<0.0001**
	Mass (g)	20 [6]	36 [19]	**0.0004**
	Mass index (g/m^2^)	11 [2]	19 [8]	**<0.0001**
LV	EDV (mL)	146 [36]	123 [50]	0.057
	EDVi (mL/m^2^)	82 [12]	69 [16]	**0.024**
	ESV (mL)	69 [22]	58 [17]	0.057
	ESVi (mL/m^2^)	37 [5]	29 [8]	**0.032**
	SV (mL)	78 [21]	64 [27]	0.065
	SVi (mL/m^2^)	43 [9]	35 [11]	**0.048**
	EF (%)	51 [6]	53 [7]	0.680
	Mass (g)	66 [29]	65 [14]	0.571
	Mass index (g/m^2^)	38 [9]	36 [4]	0.387
	Cardiac output (L/min)	4.7 [1.2]	4.5 [2.6]	0.477
	Cardiac index (L/min/m^2^)	2.71 [0.39]	2.53 [1.10]	0.632
	Contractility (mmHg/mL)	1.3 [0.3]^a^	1.8 [0.7]^b^	**0.009**

*Note*: Data is expressed as median and interquartile range [IQR]. Two‐tailed *p* values <0.05 were considered statistically significant and marked in bold.

Abbreviations: Bpm, beats per minute; CMR, cardiac magnetic resonance; EDV, end‐diastolic volume; EDVi, end‐diastolic volume indexed to body surface area; EF, ejection fraction; ESV, end‐systolic volume; ESVi, end‐systolic volume indexed to body surface area; LV, left ventricular; PH, precapillary pulmonary hypertension; RV, right ventricular; SV, stroke volume; SVi, stroke volume indexed to body surface area.

^a^ *n* = 11.

^b^ *n* = 19.

### 3.1 Kinetic energy in relation to hemodynamic indices

**FIGURE 3:**
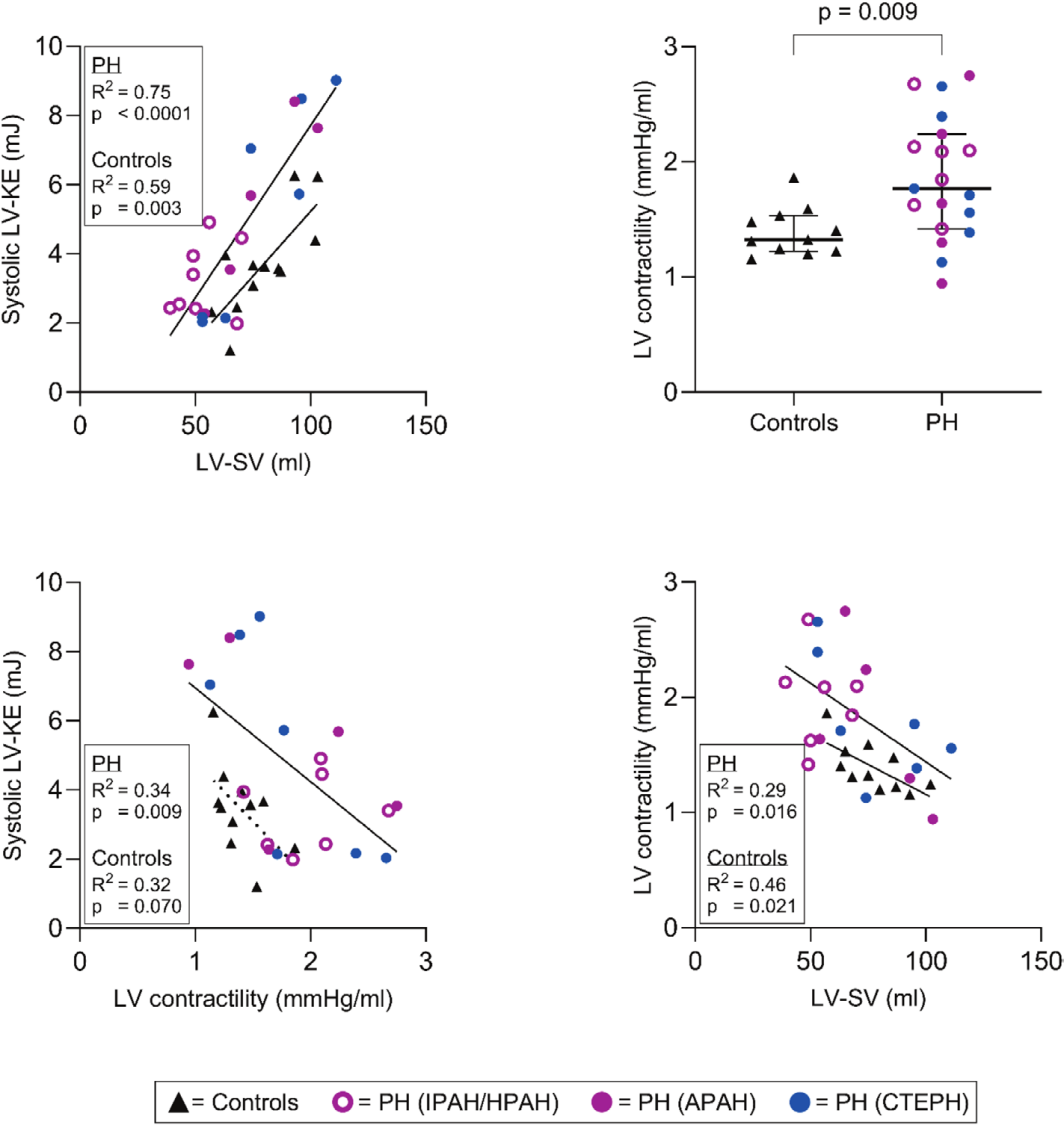
Contractility, peak systolic left ventricular (LV) kinetic energy (KE), and LV stroke volume (SV). Triangles denote healthy controls, while circles denote patients with precapillary pulmonary hypertension (PH). APAH, pulmonary arterial hypertension associated with connective tissue disease; CTEPH, chronic thromboembolic pulmonary hypertension; HPAH, hereditable pulmonary arterial hypertension; IPAH, idiopathic pulmonary arterial hypertension.

Noninvasive LV pressure volume loops were computed for 19 patients and 11 controls, since one patient and one control did not have brachial blood pressure measurements at CMR (Table 1). LV contractility was higher in patients compared to controls (Table 3, Figure 3). Systolic LV‐KE was positively associated with LV‐SV in both groups and negatively associated with LV contractility in patients, but not in controls (Table 4, Figure 3). LV contractility was negatively associated with LV‐SV in both groups.

**TABLE 4:** Linear regression analysis of KE versus hemodynamic parameters.

		Controls		PH	
		*R* ^2^ value	*p* Value	*R* ^2^ value	*p* Value
RV	Systolic KE versus PVR	**–**	**–**	0.16^b^	0.151^b^
	Systolic KE/SV versus PVR	**–**	**–**	0.01^b^	0.708^b^
	SV versus PVR	**–**	**–**	0.16^b^	0.163^b^
	Systolic KE versus SV	0.37	**0.037**	0.43	**0.002**
LV	Systolic KE versus SV	0.59	**0.003**	0.75	**<0.0001**
	Systolic KE versus contractility	0.32^a^	0.070^a^	0.34^c^	**0.009** ^c^
	Contractility versus SV	0.46^a^	**0.021** ^a^	0.29^c^	**0.016** ^c^

*Note*: Two‐tailed *p* values <0.05 were considered statistically significant and marked in bold. Abbreviations: KE, peak kinetic energy; LV, left ventricular; PH, precapillary pulmonary hypertension; PVR, pulmonary vascular resistance; RV, right ventricular; SV, stroke volume.

^a^ *n* = 11.

^b^ *n* = 14.

^c^ *n* = 19.

**FIGURE 4:**
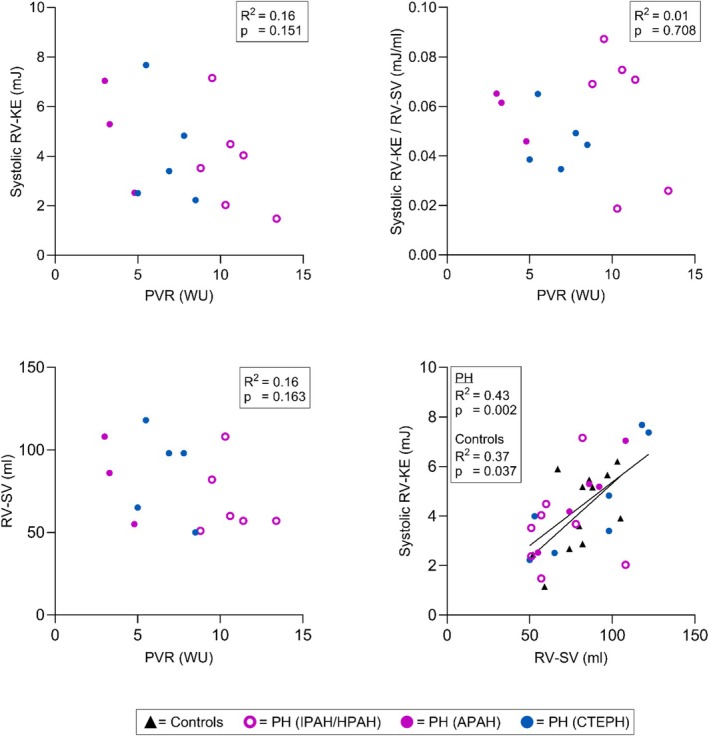
Associations between peak systolic right ventricular (RV) kinetic energy (KE), pulmonary vascular resistance (PVR), and stroke volume (SV). Triangles denote healthy controls, and circles denote patients with precapillary pulmonary hypertension (PH). APAH, pulmonary arterial hypertension associated with connective tissue disease; CTEPH, chronic thromboembolic pulmonary hypertension; HPAH, hereditable pulmonary arterial hypertension; IPAH, idiopathic pulmonary arterial hypertension.

Six patients could not be included in the PVR associations, due to significant changes in clinical status and medications (*n* = 1, 15 days between CMR and right heart catheterization), or due to more than 4 weeks between the two examinations (*n* = 5). Of the latter five patients, four underwent right heart catheterization and CMR within 76, 150, 315, and 716 days, respectively. One patient underwent diagnostic catheterization 10 years prior to CMR and had thereafter declined further catheterizations. Finally, 14 patients met the criteria for inclusion in the PVR associations. There was no association between PVR and systolic RV‐KE, RV‐SV, or systolic RV‐KE indexed for RV‐SV (Table 4, Figure 4). However, systolic RV‐KE showed a positive association to RV‐SV in both patients and controls.

### 3.2 Kinetic energy in PH compared to controls

**FIGURE 5:**
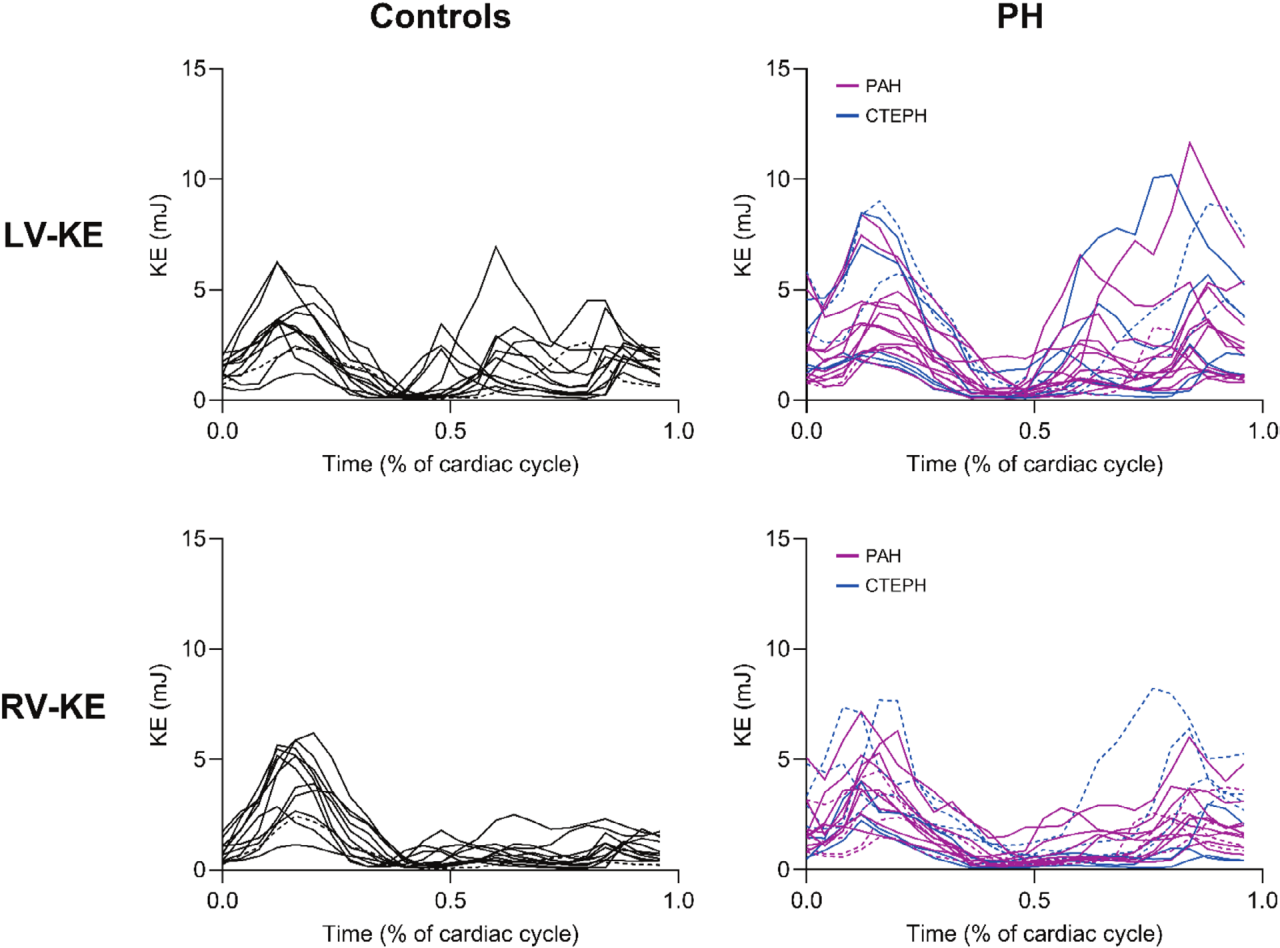
Kinetic energy (KE) time curves during the entire cardiac cycle, for all study participants. Black lines denote healthy controls, magenta lines patients with pulmonary arterial hypertension (PAH), and blue lines patients with chronic thromboembolic pulmonary hypertension (CTEPH). Dashed lines represent cases of diastolic fusion, where the early and late filling phases could not be separated.

**FIGURE 6:**
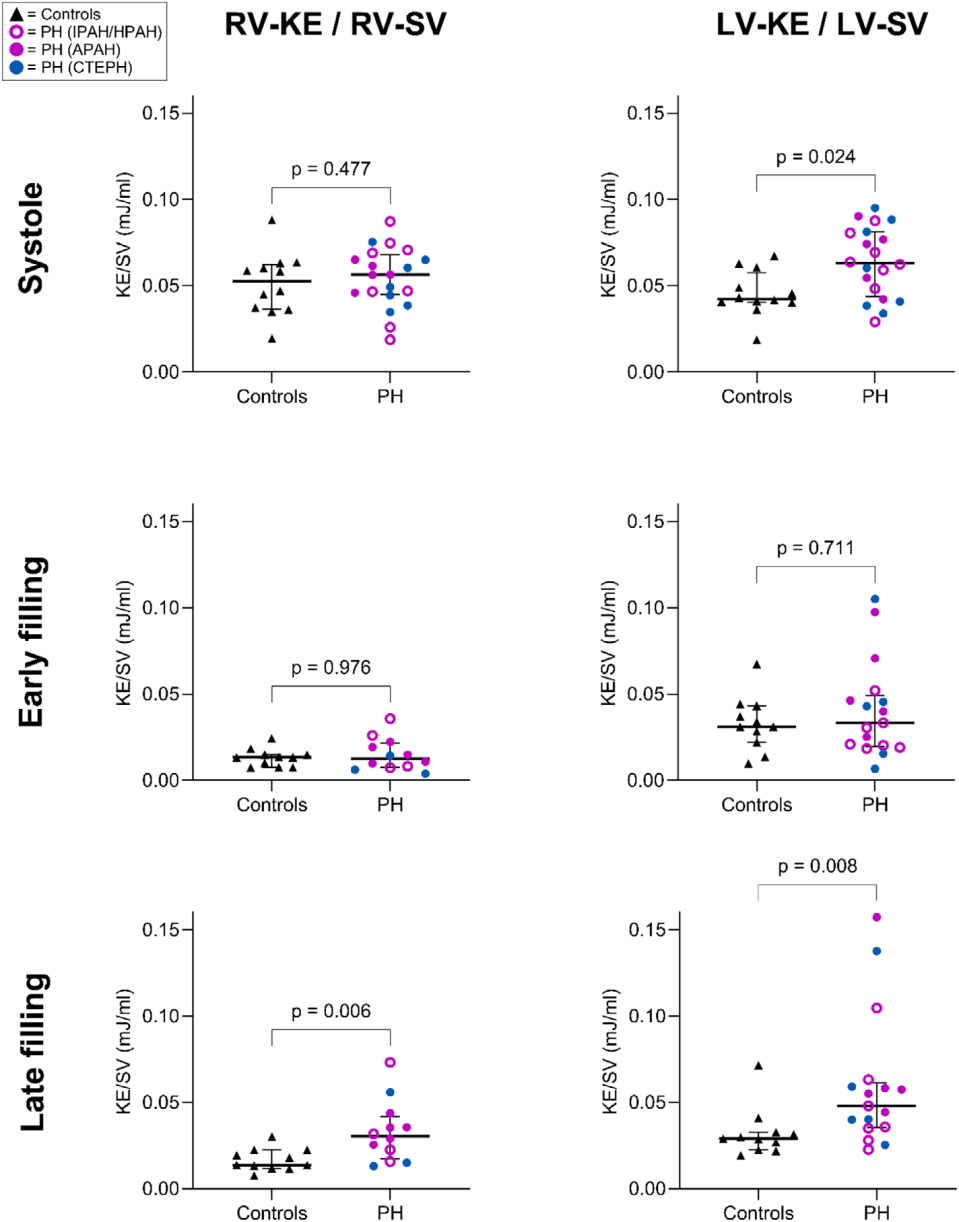
Peak kinetic energy (KE) indexed to stroke volume (SV) during systole, early filling, and late filling, in the right and left ventricle. Triangles denote healthy controls, and circles denote patients with precapillary pulmonary hypertension (PH). APAH, pulmonary arterial hypertension associated with connective tissue disease; CTEPH, chronic thromboembolic pulmonary hypertension; HPAH, hereditable pulmonary arterial hypertension; IPAH, idiopathic pulmonary arterial hypertension.

Time curves, representing KE for each participant during the entire cardiac cycle, are seen in Figure 5. Diastolic RV‐KE could be separated into early and late filling in 12 (60%) patients and 11 (92%) controls, and LV‐KE in 17 (85%) patients and 11 (92%) controls. Total diastolic RV‐KE (early and late filling treated as one continuous phase) and late filling RV‐KE, both indexed to RV‐SV, were higher in patients compared to controls (Table 5, Figure 6). There was no difference between groups during systole and early filling for the RV (Table 5, Figure 6). Total diastolic LV‐KE indexed to LV‐SV was similar between groups (Table 5). LV‐KE indexed to LV‐SV during systole and late filling was higher in patients compared to controls, while there was no difference between groups during early filling (Figure 6).

**TABLE 5:** Peak kinetic energy indexed to stroke volume, in patients with precapillary pulmonary hypertension and controls.

Peak kinetic energy indexed to SV (mJ/mL)		Controls (*n* = 12)	PH (*n* = 20)	*p* Value
RV	Systole	0.053 [0.024]	0.056 [0.021]	0.477
	Diastole, total	0.016 [0.008]	0.036 [0.022]	**<0.0001**
	Early filling	0.013 [0.006]^a^	0.013 [0.012]^b^	0.976
	Late filling	0.014 [0.008]^a^	0.030 [0.017]^b^	**0.006**
LV	Systole	0.042 [0.011]	0.063 [0.034]	**0.024**
	Diastole, total	0.036 [0.014]	0.052 [0.034]	0.053
	Early filling	0.031 [0.015]^a^	0.033 [0.026]^c^	0.711
	Late filling	0.029 [0.007]^a^	0.048 [0.023]^c^	**0.008**

*Note*: Data is expressed as median and interquartile range [IQR]. Two‐tailed *p* values <0.05 were considered statistically significant and marked in bold.

Abbreviations: LV, left ventricular; PH, precapillary pulmonary hypertension; RV, right ventricular; SV, stroke volume.

^a^ *n* = 11.

^b^ *n* = 12.

^c^ *n* = 17.

Mean RV‐KE and LV‐KE showed similar results as peak KE (Table S2, Figures S2 and S3). Peak KE in absolute values is reported in Table S3. Peak KE indexed to end‐diastolic volume (EDV) is reported in Table S4.

## 4 DISCUSSION

We found that patients with PH exhibited higher systolic LV‐KE at a given SV than controls, as well as higher heart rate and LV contractility, suggesting sympathetic upregulation as a possible driving mechanism behind increased systolic LV‐KE. Furthermore, systolic RV‐KE was associated with RV‐SV in patients with PH as well as in controls. Yet, PVR was not associated with systolic RV‐KE, suggesting that the RV may still be able to compensate for the increased afterload. Finally, we found an augmented late diastolic KE in PH, suggesting an increased reliance on the atrial kick to fill both ventricles in this cohort.

### 4.1 Physiological factors contributing to altered KE

Patients with PH typically have smaller LV‐SV than healthy individuals. To take the mass effect of the moving blood into account, we chose to index LV‐KE to LV‐SV. Our finding that systolic LV‐KE in absolute numbers was similar between groups, but higher in patients when indexed to LV‐SV, suggests that the velocities during systole were increased with a concomitant decrease in stroke volume. Increased velocities seen in patients during systole could be either due to the blood having less time to decelerate from diastole to systole because of a higher heart rate, or that the LV accelerates the blood more during systole per ejected volume in patients compared to controls. A higher acceleration could indicate increased LV contractility. Noninvasive LV pressure volume loops indeed showed higher contractility among patients with PH compared to controls. The relationship between LV end‐systolic volume and pressure may also be accentuated by septal bowing that further reduces the LV end‐systolic volume. It is so far unknown to what extent abnormal septal movement affects noninvasive analysis of contractility.

**FIGURE 7:**
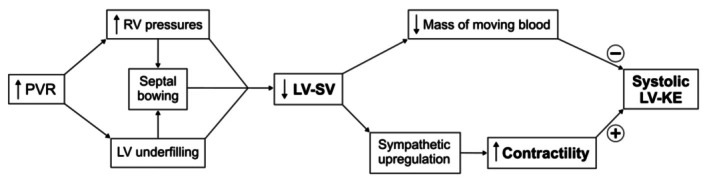
A schematic illustration of how increased pulmonary vascular resistance (PVR) and the resulting left ventricular (LV) underfilling may affect systolic LV kinetic energy (KE). The reduced LV stroke volume (SV) may both decrease the systolic LV‐KE by a smaller mass of moving blood, but also increase the KE by sympathetic upregulation and higher contractility. RV, right ventricular.

The patients in our study showed a tendency towards higher systolic LV‐KE for a given LV contractility, compared with controls. Patients with a low systolic LV‐KE and low LV‐SV also had the highest LV contractility. A high contractility suggests that the LV systolic function was intrinsically normal, since contractility would be expected to decrease in a failing LV. The lower LV‐KE in the patients with a high contractility may instead be due to an underfilled LV (Sjogren et al., 2021). This, as depicted in Figure 7, could explain the counterintuitive negative relationship between systolic LV‐KE and contractility—a patient with an underfilled LV (with a reduction in both LV‐SV and systolic LV‐KE) may have a lower cardiac output, leading to sympathetic upregulation and increased contractility. Thus, the negative relationship between systolic LV‐KE and contractility is likely influenced by the LV‐SV. Indeed, we found that differences in LV‐SV are strongly associated with both systolic LV‐KE and LV contractility.

Systolic RV‐KE and PVR showed no association, neither did RV‐SV and PVR. The latter could suggest that the RV was still able to compensate for the increased PVR (Humbert et al., 2022) and may explain the similar systolic RV‐KE seen in patients and controls. If the patients had been in decompensated RV failure, the RV would succumb to the increased afterload. In this scenario, forward motion of the blood would decrease, incurring—at least theoretically—a reduction in systolic RV‐KE. Yet, studies remain to assess RV‐KE in the late stages of PAH.

### 4.2 RV‐KE

We found a higher diastolic RV‐KE in patients with PH compared to controls, possibly driven by increased stiffness and impaired relaxation of the RV as previously reported in PH (Rain et al., 2013; Trip et al., 2015; Wessels et al., 2022). With impaired RV relaxation, the dependency of the atrial kick on ventricular filling is likely substantial (Alenezi et al., 2018; Heywood et al., 1990; Willens et al., 2008), thereby increasing the right atrial stroke work (Wessels et al., 2022).

We found no difference in RV‐KE indexed to either RV‐EDV or RV‐SV during early filling between groups. Our results differ from a previous assessment of early filling KE, where RV‐KE indexed to RV‐EDV was lower in PH than controls (Zhao et al., 2022). However, the previous study included patients with shunts and thus, EDV may have been larger in some patients. As we excluded patients with shunts, differences in EDV and hemodynamic state may explain the dissimilarity of the results.

Early and late filling RV‐KE could only be separated in 60% of our patient population; the remaining exhibited fusion of the two filling phases. Fusion of early and late filling is commonly understood to be related to increased heart rate (Chung et al., 2004; Nagueh et al., 2016), and this was seen in the present PH population. However, 25% of the patients had fusion of E and A waves only in the RV, with retained diastasis in the LV. This indicates that in patients with PH, diastolic filling is affected differently in the RV compared to the LV, and that an increased heart rate is not the only explanation for diastolic fusion.

Systolic RV‐KE was similar between patients with PH and controls in our study, supporting results from Zhao et al. (2022). The similar systolic RV‐KE—despite dissimilar diastolic RV‐KE between patients and controls in our study—may indicate that diastolic function is affected at an earlier stage of the disease than the systolic function is.

### 4.3 LV‐KE

Late filling LV‐KE indexed to LV‐SV was higher in patients than controls in our study, likely due to patients exhibiting a higher heart rate. The higher heart rate leads to a shorter duration of diastole, which could potentially necessitate higher late filling velocities for sufficient volume to enter the small ventricle before the next ejection. In addition, the LV early filling pressure gradient is decreased in PH, suggestive of impaired LV suction and, potentially, a result of a decreased LV preload (Gurudevan et al., 2007). With impaired early filling due to decreased preload, the atrial kick may be crucial to filling the LV sufficiently in PH.

Early filling LV‐KE was similar between groups in our study, suggesting unaltered relaxation and an LV without intrinsic myocardial disease. In contrast, Zhao et al. (2022) reported lower early filling LV‐KE in patients with PH than controls, and no difference in LV‐EDV indexed to body surface area between groups. Our study population differs from Zhao et al. (2022) where patients with left atrial filling pressure ≥ 15 mmHg and patients with untreated shunts were included. Hence, some of the patients in Zhao et al. could not strictly be categorized as having precapillary PH. In our study, no patients had increased left atrial filling pressures or present shunts, and LV‐EDV indexed to body surface area was lower in PH than controls. The lower early filling LV‐KE seen in patients by Zhao et al. might thus, to some part, be explained by indexing for LV‐EDV, different patient characteristics, and hemodynamics.

### 4.4 Limitations

We included both patients with PAH and CTEPH, since the intracardiac pressure conditions are expected to be similar in both groups. However, larger sample sizes would be needed to further assess any subgroup differences. Overall, the sample size was relatively small, especially in the analyses of PVR (where only 14 patients could be included) and in separating diastolic KE into early and late filling. No a priori power calculation could be done as this is the first study addressing how PVR and LV contractility affect KE, and our study may therefore be underpowered. However, our exploratory study may provide a framework for future, larger studies to build upon, including subgroup analyses of pump physiology and of the prognostic utility of KE assessment in patients with PH. Additionally, larger studies would allow adjustment for other physiological variables such as heart rate and blood pressure, which could clarify independent associations. Images from patients with shortness of breath may have breathing artifacts, increasing the risk of poor 4D quality and thus exclusion from the study. Noninvasive PV loops have been validated in a porcine model and in healthy subjects (Seemann et al., 2019; Sjöberg et al., 2021), but not in patients with PH. Interventricular dependence and pressure differences may cause septal bowing in PH, where the interventricular septum deviates to the left. Thus, septal bowing may alter end‐systolic volumes, thus potentially affecting the estimation of LV contractility in some PH patients.

## 5 CONCLUSIONS

Patients with PH exhibited higher systolic LV‐KE at a given SV than controls, as well as higher heart rate and LV contractility, suggesting sympathetic upregulation as a possible driving mechanism behind increased systolic LV‐KE. PVR was not associated with systolic RV‐KE, suggesting that the RV may still be able to compensate for the increased afterload. The augmented late diastolic KE suggests an increased reliance on the atrial kick to fill the ventricles in patients with PH.

## AUTHOR CONTRIBUTIONS

JT, MC, HA, and EO conceived and designed research. GR performed clinical evaluation for experimental analysis. EB, KP, JT, and EO analyzed data. EB, KP, JT, BK, PMA, HA, MC, and EO interpreted results of experiments. EB prepared figures. EB, BK, and EO drafted manuscript. EB, KP, BK, JT, PMA, MC, GR, HA, and EO Edited and revised manuscript and approved final version of manuscript.

## FUNDING INFORMATION

This study was funded by Swedish Research Council, Stockholm, Sweden [grant number 2021‐02779]; Region Skåne (ALF), Lund Sweden; Swedish Society of Medicine, Stockholm, Sweden [grant number SLS‐961558 and SLS‐999997]; Swedish Heart and Lung Foundation, Stockholm, Sweden [grant numbers 20190576, 20210337, 20220449, and 20230550]; Crafoord Foundation, Lund, Sweden [grant number 200681 and 20230611]; Southern Healthcare Region of Sweden; Faculty of Medicine, Lund University; Skåne University Hospital Foundations.

## CONFLICT OF INTEREST STATEMENT

G.R. reports personal lecture fees from AOP Health/Orpha Care, Janssen, MSD, and Nordic Infucare outside the submitted work; and is or has been a primary investigator or co‐investigator in clinical PAH trials for Acceleron, Actelion Pharmaceuticals Sweden AB, Acceleron, Bayer HealthCare, GlaxoSmithKline, Janssen, MSD, Pfizer, and United Therapeutics and in clinical heart transplantation immunosuppression trials for Novartis. The companies had no role in the data collection, analysis, and interpretation and had no right in disapproving of the manuscript.

## ETHICS STATEMENT

All subjects gave written informed consent to participate in the study prior to examination, and the Regional Ethical Committee in Lund, Sweden, approved the use of data for research (application numbers 2010/114, 2010/248, 2011/777). The study complied with the Declaration of Helsinki.

## Supporting information


Appendix S1.

## Data Availability

The datasets used and/or analyzed during the current study are available from the corresponding author upon reasonable request.
