# Supplementary material for: Increased contractility affects left ventricular kinetic energy in pulmonary hypertension
Source: Physiol Rep. 2025 Sep 12;13(17):e70563. doi: 10.14814/phy2.70563 (PMC12431581; doi:10.14814/phy2.70563)
Supplement: Supplementary file 1 — Appendix S1. [file PHY2-13-e70563-s001.pdf]

Supplementary materials

Definition of cardiac phases from 4D flow

End diastole for KE analysis was defined as the first time frame in the 4D flow data. The pulmonary artery and ascending aorta were delineated in all time frames in standard 2D phase contrast images, with delineations superposed to 4D flow. End systole was defined from the 4D flow curves of the pulmonary artery for the RV and of the ascending aorta for the LV. A line was extrapolated from the systolic downslope of 4D flow in each vessel respectively, going through two points at 75% and 25% of maximum amplitude, with end systole for KE analysis determined as the point where the extrapolated line reached zero (Figure 2A).

The transition from early to late filling during diastole was defined using transtricuspid and transmitral flow curves at the approximate level of the valve leaflets, as previously described [1]. A line was extrapolated from the late filling upslope of 4D flow, going through two points at 75% and 25% of maximum amplitude, with beginning of late filling determined as the point where the extrapolated line reached zero, in the cases with flow curves reaching zero in diastasis (Figure 2B). In the cases where flow curves did not reach zero, the lowest point of the flow curve between early and late filling defined the start of late filling diastole (Figure 2C). In cases of fusion with no nadir between early and late filling, only total diastolic KE was reported, where early and late filling are treated as one continuous phase. Total diastolic KE (early and late filling treated as one continuous phase) is reported from all participants, while early and late filling values are only reported where the two phases could be separated.

Supplementary Table 1. Typical 4D flow image parameters

**Parameter**	
Readout strategy	Cartesian
Respiratory gating	Respiratory gating in 20 cases (15 patients, 5 controls), 12 without (5 patients, 7 controls).
Velocity encoding (VENC) (cm/s)	100 (n=22), 150 (n=6), 250 (n=4)
Echo time (ms)	2.8-3.5
Repetition time (ms)	5.8
Flip angle (°)	15 (n=27, post contrast), 8 (n=5, no contrast)
Bandwidth/pixel (Hz)	558
Field of view (mm^3)	288 × 240 × 162
Matrix size (readout × phase × slice)	96 × 80 × 54
Acquired spatial resolution (mm^3)	3 × 3 × 3
Reconstructed spatial resolution (mm^3)	3 × 3 × 3
Temporal segmentation factor	2
Acquired temporal resolution (ms)	46
Reconstructed temporal resolution (ms)	16-29 (40 phases per cardiac cycle)
Acceleration methods	GRAPPA (phase × slice: 2×2 or 3x1)<br>partial Fourier (phase × slice: 6/8 × 6/8)
Postprocessing	Maxwell correction, background correction, phase unwrapping.

Supplementary Table 2: Mean kinetic energy (absolute and indexed to stroke volume) in patients with precapillary pulmonary hypertension and controls.

	Mean kinetic energy in absolute values (mJ) and indexed to SV		**Controls (n=12)**	**PH (n=20)**	**p-values**
RV	Absolute values (mJ)	Systole	2.0 [1.4]	2.0 [1.2]	0.716
		Diastole, total	0.7 [0.4]	1.4 [0.8]	***0.005***
		Early filling	0.6 [0.4] [a]	0.6 [0.8] [b]	0.786
		Late filling	0.9 [0.6] [a]	1.7 [1.1] [b]	***0.037***
	Indexed to SV (mJ/ml)	Systole	0.02 [0.01]	0.03 [0.01]	0.272
		Diastole, total	0.008 [0.003]	0.02 [0.006]	***0.002***
		Early filling	0.007 [0.004] [a]	0.009 [0.007] [b]	0.347
		Late filling	0.008 [0.008] [a]	0.02 [0.01] [b]	***0.007***
LV	Absolute values (mJ)	Systole	1.7 [0.8]	2.2 [2.1]	0.387
		Diastole, total	1.2 [0.7]	1.5 [2.0]	0.454
		Early filling	1.1 [0.6] [a]	0.8 [1.5] [c]	0.926
		Late filling	1.7 [0.7] [a]	2.4 [2.5] [c]	0.378
	Indexed to SV (mJ/ml)	Systole	0.02 [0.007]	0.03 [0.02]	***0.001***
		Diastole, total	0.02 [0.008]	0.02 [0.02]	***0.029***
		Early filling	0.01 [0.008] [a]	0.02 [0.02] [c]	0.208
		Late filling	0.02 [0.007] [a]	0.03 [0.02] [c]	***0.013***

Data expressed as median and interquartile range [IQR]. LV, left ventricular; PH, precapillary pulmonary hypertension; RV, right ventricular; SV, stroke volume.
a: n=11; b: n=12; c: n=17.

Supplementary Table 3: Peak kinetic energy (absolute values) in patients with precapillary pulmonary hypertension and controls.

Peak kinetic energy in absolute values (mJ)		**Controls (n=12)**	**PH (n=20)**	**p-value**
RV	Systole	4.5 [2.7]	4.0 [2.7]	0.863
	Diastole, total	1.3 [0.8]	2.7 [1.5]	***0.0004***
	Early filling	1.1 [0.4] [a]	0.8 [1.3] [b]	0.695
	Late filling	1.3 [0.7] [a]	2.4 [1.6] [b]	***0.037***
LV	Systole	3.6 [1.1]	3.7 [3.7]	0.744
	Diastole, total	2.8 [1.2]	3.5 [3.3]	0.477
	Early filling	2.5 [1.0] [a]	2.1 [2.9] [c]	0.643
	Late filling	2.5 [0.5] [a]	3.4 [3.2] [c]	0.264

Data is expressed as median and interquartile range [IQR]. LV, left ventricular; PH, precapillary pulmonary hypertension; RV, right ventricular; SV, stroke volume.
a: n=11; b: n=12; c: n=17.

Supplementary Table 4: Peak kinetic energy (indexed to end-diastolic volume) in patients with precapillary pulmonary hypertension and controls.

Peak kinetic energy indexed to EDV (mJ/ml)		**Controls (n=12)**	**PH (n=20)**	**p-value**
RV	Systole	0.03 [0.01]	0.02 [0.01]	0.195
	Diastole, total	0.008 [0.004]	0.02 [0.008]	***0.003***
	Early filling	0.007 [0.003] [a]	0.007 [0.007] [b]	0.608
	Late filling	0.007 [0.005] [a]	0.01 [0.01] [b]	0.079
LV	Systole	0.02 [0.008]	0.03 [0.02]	***0.013***
	Diastole, total	0.02 [0.006]	0.03 [0.02]	***0.036***
	Early filling	0.02 [0.006] [a]	0.02 [0.02] [c]	0.643
	Late filling	0.02 [0.003] [a]	0.02 [0.01] [c]	***0.019***

Data is expressed as median and interquartile range [IQR]. EDV, end-diastolic volume; LV, left ventricular; PH, precapillary pulmonary hypertension; RV, right ventricular; SV, stroke volume.

a: n=11; b: n=12; c: n=17.

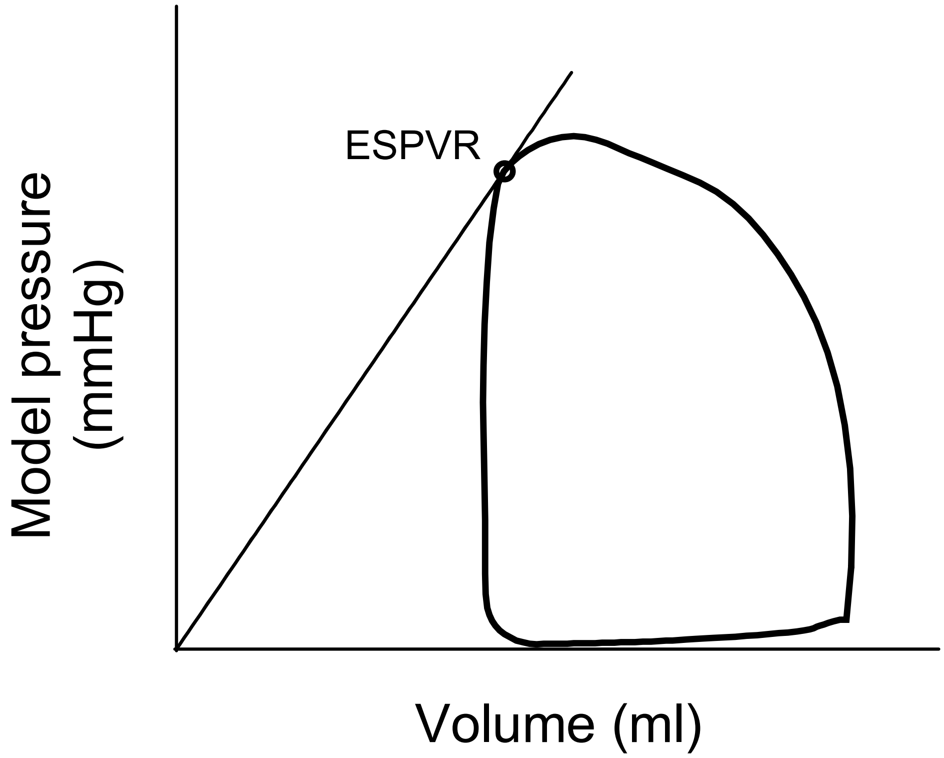

Supplementary Figure 1. A left ventricular pressure volume loop from a patient with precapillary pulmonary hypertension. The pressure volume loop was generated using a non-invasive elastance model [2, 3], and contractility was computed as the slope of the end-systolic pressure volume relationship (ESPVR).

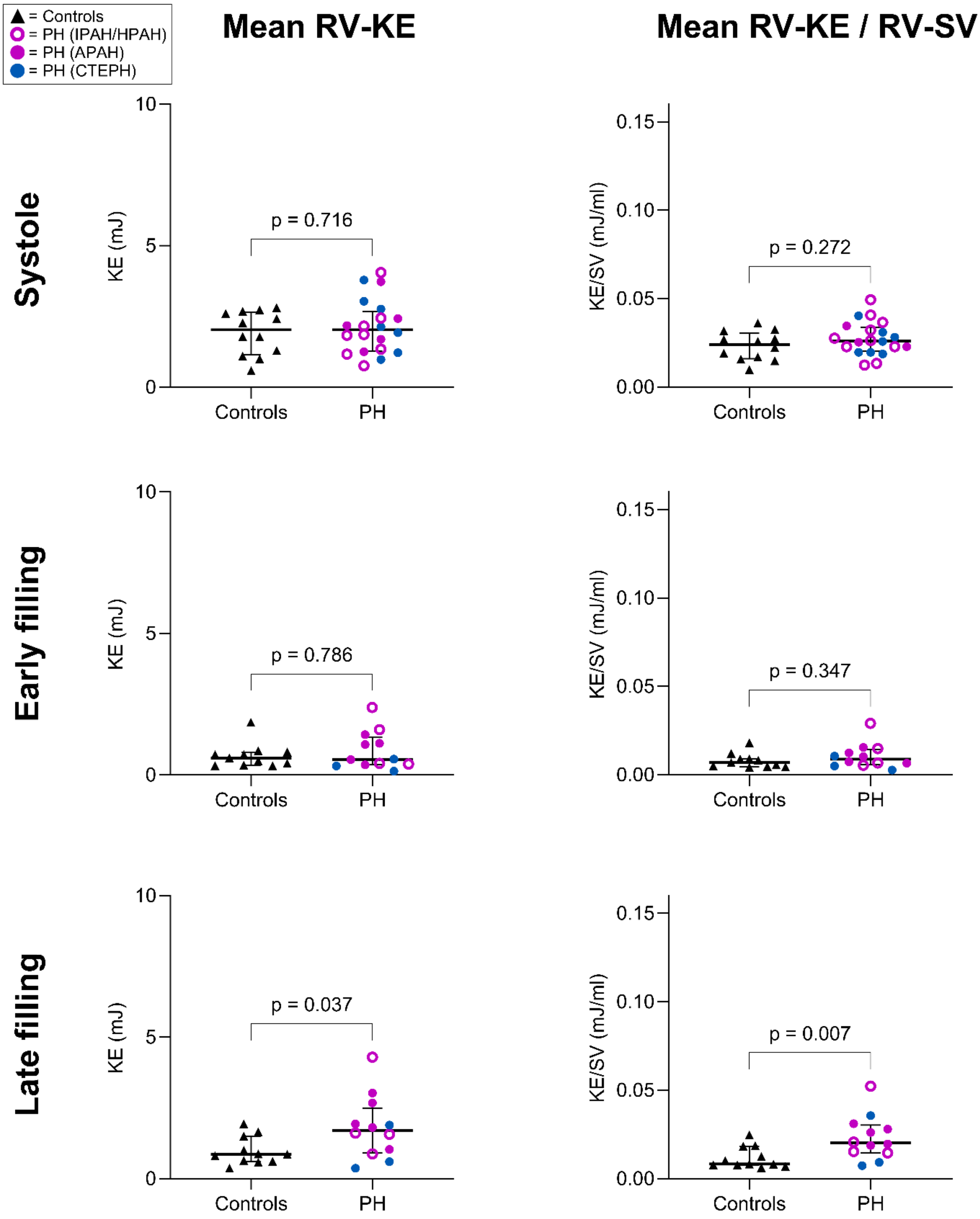

Supplementary Figure 2. Mean right ventricular (RV) kinetic energy (KE) during systole, early filling and late filling, in absolute values (left) and indexed to RV stroke volume (SV) (right). Triangles denote healthy controls, circles patients with precapillary pulmonary hypertension (PH). APAH, pulmonary arterial hypertension associated with connective tissue disease; CTEPH, chronic thromboembolic pulmonary hypertension; HPAH, hereditable pulmonary arterial hypertension; IPAH, idiopathic pulmonary arterial hypertension.

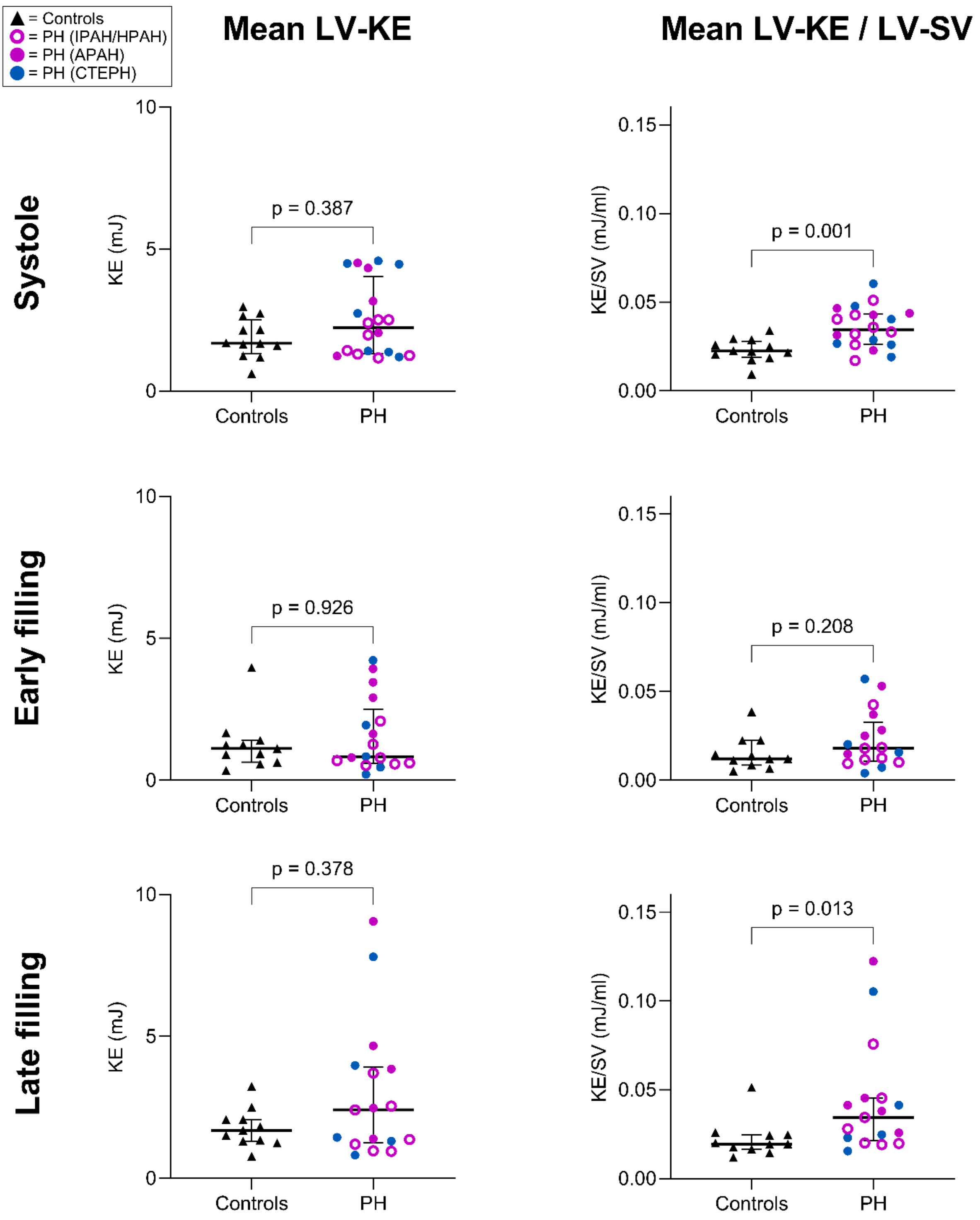

Supplementary Figure 3. Mean left ventricular (LV) kinetic energy (KE) during systole, early filling and late filling, in absolute values (left) and indexed to RV stroke volume (SV) (right). Triangles denote healthy controls, circles patients with precapillary pulmonary hypertension (PH). APAH, pulmonary arterial hypertension associated with connective tissue disease; CTEPH, chronic thromboembolic pulmonary hypertension; HPAH, hereditable pulmonary arterial hypertension; IPAH, idiopathic pulmonary arterial hypertension.

References Supplementary Materials

[1] Toger J, Kanski M, Carlsson M, Kovacs SJ, Soderlind G, Arheden H, et al. Vortex ring formation in the left ventricle of the heart: analysis by 4D flow MRI and Lagrangian coherent structures. Ann Biomed Eng. 2012;40(12):2652-62.
[2] Seemann F, Arvidsson P, Nordlund D, Kopic S, Carlsson M, Arheden H, et al. Noninvasive Quantification of Pressure-Volume Loops From Brachial Pressure and Cardiovascular Magnetic Resonance. Circ Cardiovasc Imaging. 2019;12(1):e008493.
[3] Sjöberg P, Seemann F, Arheden H, Heiberg E. Non-invasive quantification of pressure-volume loops from cardiovascular magnetic resonance at rest and during dobutamine stress. Clin Physiol Funct Imaging. 2021;41(5):467-70.
